# External funding to strengthen capacity for research in low-income and middle-income countries: exigence, excellence and equity

**DOI:** 10.1136/bmjgh-2019-002212

**Published:** 2020-03-17

**Authors:** Dermot Maher, Abraham Aseffa, Simon Kay, Marta Tufet Bayona

**Affiliations:** 1 Research Capacity Strengthening, Special Programme for Research and Training in Tropical Diseases, Geneva, Switzerland; 2 Armauer Hansen Research Institute (AHRI), Kolfe Keranyo, Jimma Road, ALERT Compound, Addis Ababa, Ethiopia, Addis Ababa, Ethiopia; 3 International Operations and Partnerships, Wellcome Trust, London, UK; 4 United Kingdom Collaborative on Development Research, c/o Wellcome Trust, London, UK

**Keywords:** health research, capacity, equity, excellence

**Summary box**

- Strengthening capacity for research is of vital relevance to global health since research is fundamental to the improvement of health everywhere, but the capacity to do research varies enormously between countries.
- External donors broadly agree on the exigence to support national efforts to strengthen the capacity for health research in low-income and middle-income countries.
- Current levels of external funding may support the concentration of activities in pursuit of excellence in some countries without achieving the aims of equity to ensure all countries can benefit from producing research.
- Key elements for debate as external donors and partner countries pursue the benefits of excellence and equity include: (1) the need for evidence-based decision making, (2) the promotion of standardised collection and open reporting of data, (3) the level of funding which can avoid competition between excellence and equity, (4) revisiting what ‘excellence’ means, and (5) the implications of a shift to local leadership and knowledge in driving development practice.

## Introduction

As research is fundamental to the improvement of health everywhere, and the achievement of Universal Health Coverage (UHC), all countries irrespective of their income level should be producers of research as well as consumers.^1^ This is particularly true of implementation research, which depends on the creativity and skills of researchers close to the supply of, and demand for, health services to contribute to improved UHC coverage.^1^ However, there is a great disparity between regions and countries in their capacity to produce research. For example, Africa is home to 15% of the world’s population and 5% of the world’s gross domestic product but accounts for just 1.3% of global health research publications.^2^ Ranking the countries of sub-Saharan Africa by the number of researchers per million inhabitants (as an indicator of research capacity), South Africa is top (818), while Burundi, the Central African Republic, the Gambia, Lesotho and Zambia are at the bottom (each with <50).^3^ As an indication of the scale of the need to strengthen capacity, for Africa to achieve even the world average for the number of researchers per head of population, it will need to rapidly train one million new PhDs.^4^

Advocates have long championed building and sustaining research capacity as a leading strategy to overcome health disparities worldwide.^5^ There is now increasing recognition of the lack of research capacity as a primary determinant of poverty in low-income and middle-income countries (LMICs).^6^ Building and sustaining research capacity involves a wide range of activities, including developing the institutional base, research infrastructure, relevant training programmes, career development pathways, research portfolio, regulatory environment and networks. Although responsibility for building and strengthening research capacity should primarily lie with national governments, funding usually depends on a mix of domestic investment and external support through a wide range of initiatives^7^ and funding modalities.^8^ External funding may be linked to support and investment at the national level, as well as to support for institutions and individuals. An illustrative example is the multifunder Science Granting Councils Initiative.^9^ Ethical best practice involves external funders working with partner countries to ensure that they support and complement national strategies, plans and activities.

In this perspective, we consider how the external donor response to this exigence, that is, the need to strengthen research capacity in LMICs, should reflect considerations of excellence and equity. Funders often use excellence as a key criterion in making awards usually through open competition among individuals or institutions, with the aim of enabling highly performing researchers to undertake high-quality research. However, funders should also contribute to strengthened research capacity in ways that take into account equity, with the aim of helping to ensure that all countries obtain the benefits of producing research. Excellence and equity may be seen as competing considerations if decisions to award funds are mainly based on excellence at the expense of equity. This results in a concentration of external support in particular countries, even though all countries stand to benefit from the strengthened capacity for research. Funders need to address both excellence and equity as linked, rather than competing, considerations as part of a full response to the need to strengthen capacity in LMICs.

Although the focus of this paper is on LMICs in sub-Saharan Africa, the issues are also likely to be relevant to LMICs in other regions. The arguments in this perspective apply mainly to clinical and implementation research in the near-term to mid-term perspective, but may also apply to basic biomedical research in the longer-term perspective.

## Countries caught in a Catch-22 of a lack of competitiveness and lack of capacity

Selection policies for external funding of activities to strengthen research capacity are often primarily based on excellence in assessing the quality of the proposed activities. This tends to result in concentrated support for activities in a particular set of institutions in a particular set of countries. An example of a concentration of external funding in a set of countries is shown by the results of mapping externally funded international postgraduate training at institutions in sub-Saharan Africa.^10^ The eastern and southern Africa regions were most competitive and therefore received more external funder support, which was concentrated in six countries: Ethiopia, Kenya, Nigeria, South Africa, Tanzania and Uganda.^10^ A group of countries (Cape Verde, Central African Republic, Djibouti, Equatorial Guinea, Eritrea, Lesotho, Liberia, Namibia, Sao Tome and Principe, and Somalia) received no external funding.^10^

All countries could benefit from support to strengthen research capacity as part of the development of national research systems and enable them to obtain the health and development benefits of research. However, a wide range of institutions and countries tend not to receive external support because they are not competitive (in terms of winning awards based on excellence). They, therefore, remain locked in a Catch-22 of a lack of competitiveness and lack of capacity. If the goal is to generate the greatest health and development gains through research, the question arises as to how to optimise the extent and distribution of support through consideration of both excellence and equity to achieve this goal. The occurrence of the 2014–2015 Ebola outbreak ‘of unprecedented magnitude in a setting of limited capacity’ led to renewed awareness of the need for national governments of LMICs and development partners to invest in clinical research capacity, with opportunities identified for investments through two trust fund mechanisms at the World Bank, the Pandemic Emergency Financing Facility and the Coalition for Epidemic Preparedness Innovations.^11^ Investments in clinical research capacity alone are unlikely to yield the expected return unless accompanied by the implementation research on ensuring that the health products reach all those who could benefit from them. As implementation research is often context-specific, all countries would benefit from the strengthened local capacity to solve local health problems.

## How to respond to the exigence to strengthen capacity taking into account excellence and equity

External funders generally agree on the need to contribute to strengthening research capacity in their partner countries among the LMICs, as recognised, for example, by ESSENCE on Health Research (an initiative of funding agencies)^12^ and by the United Kingdom Collaborative for Development Research (UKCDR), through the Research Capacity Strengthening Group.^13^*This agreed exigence* is a good starting point for an open debate among donors and partner countries on how external funding can contribute to strengthened research capacity by enabling high-quality research (ie, by taking into account excellence) and by enabling all countries to obtain the benefits of producing research (ie, by taking into account equity). This debate should take into consideration at least five key points. First, we need to learn lessons from previous attempts at promoting excellence and equity, including the need for relevant evaluation indicators. For example, under the African Institutions Initiative funded by the Wellcome Trust, the consortia included links between universities with greater and lesser degrees of established research capacity, requiring indicators of evaluation other than those based on research quality.^14^

Second, the debate should be informed by good data on what is often a complex landscape of external funding in support of national governments external funding to strengthen research capacity. Examples of work in this area include the mapping of externally funded international postgraduate training at institutions in sub-Saharan Africa^10^ and the global review of health research capacity strengthening schemes funded by external donors.^15^ These initiatives have paved the way for the International Vaccines Task Force recommendation to develop ‘a mechanism that permits a thorough review of current and planned investments in research capacity strengthening’,^11^ which is now under discussion among the WHO Global R&D Observatory, ESSENCE initiative on health research, and World RePORT (hosted by the US National Institutes of Health). Standardised collection and reporting of data would help to avoid overlap and promote synergy between funders, and inform dialogue between external funders and partner countries on strategic issues. Collecting and analysing data on the distribution of external funding among countries in the region would also provide the first step in accountability for ensuring progress towards equity. The development of an accountability mechanism is important that reflects the principle of mutual accountability of donors and partners in the 2005 Paris Declaration on Aid Effectiveness.^16^

Third, the level of external funding should be sufficient to achieve the two aims of enabling high-quality research (as determined by excellence) and enabling all countries to obtain the benefits of producing research (by taking into account equity), that is, external funding for one aim should not be at the expense of the other. Those countries that have made progress in strengthening research capacity need further external support to build on the progress they have achieved. At the same time, external funders should support those countries trapped by the lack of competitiveness and lack of capacity, enabling them also to obtain the benefits of producing research and to contribute to global health security through enhanced preparedness against epidemic threats.

Fourth, the debate should reflect the ongoing discussion on defining and measuring excellence in science and assessing its utility.^17^ Excellence may not be an excellent measure of research quality unless it embraces local context, relevance and societal impact, and therefore by extension, the distribution of impact in terms of equity.

Fifth, the debate should be framed by a shift towards local leadership and knowledge in driving development practice. Given the importance of an African-led development agenda for sustained impact, maximising the contribution of external funding to strengthened research capacity by taking into account excellence and equity may require consideration of new models of support for organisational capacity development which reflect this shift.^18^

## Conclusion

In response to the exigence to the strengthen capacity in LMICs, funders should address excellence and equity as linked, rather than competing, considerations to build strong national research systems, thereby maximising the contribution of research to health and development gains and to global health security.
